# Comparison of fracture resistance in endodontically treated premolars with extensive MOD cavities restored using different materials: An*in vitro*study

**DOI:** 10.6026/973206300223017

**Published:** 2026-05-31

**Authors:** Ningthoukhongjam Rati Devi, Jenny Atom, Moirangthem Niteshore Singh, Nikita Kangabam, Kangjam Gunadhar Singh, Leishangthem Sophia Devi Tutor, Bebika Devi Thoudam

**Affiliations:** 1 Department of Conservative Dentistry and Endodontics, Dental College, Jawaharlal Nehru Institute of Medical Sciences, Imphal East 795005, Manipur, India; 2 Department of Conservative Dentistry and Endodontic, Dental College, RIMS, Imphal West, Manipur, India; 3 Department of ENT, Shija Academy of Health Sciences, Langol, Manipur, India; 4 Department of Public Health Dentistry, Dental College, RIMS, Imphal, Manipur, India; 5 Department of Periodontology, Dental college, Jawaharlal Nehru Institute of Medical Sciences, Imphal East, Manipur, India

**Keywords:** Fracture resistance, endodontically treated teeth, Mesio-occluso-distal (MOD) cavity, restorative materials, universal testing machine

## Abstract

Endodontically treated teeth with extensive coronal destruction are highly susceptible to fracture, making the selection of restorative material critical. Therefore, it is of interest to evaluate the fracture resistance of sixty maxillary premolars with standardized Mesio-occluso-distal (MOD) cavities restored using different materials. Specimens were divided into six groups and subjected to compressive loading using a universal testing machine, with fracture patterns analyzed under stereomicroscopy. Intact teeth showed the highest fracture resistance, while unrestored teeth showed the lowest; Multicore Flow and Ketac Molar groups demonstrated significantly higher resistance compared to amalgam and sandwich technique groups. Thus, dual-cure composite and glass ionomer cement provided superior reinforcement of weakened tooth structure, highlighting their clinical relevance.

## Background:

Maintaining and functionally restoring endodontically treated teeth is one of the most challenging clinically important issues in the modern restorative dentistry. The third most common cause of tooth loss after dental caries and periodontal disease has been reported to be tooth fracture and the endodontically treated teeth have been found to be much more susceptible to fracture than their vital counterparts [1,2]. This compromised susceptibility can be explained by a cumulative structural compromise due to a carious destruction, prior restorative procedures, cuspal support preparation and the loss of the protective roof of the pulp chamber due to this cumulative structural compromise [3, 4]. Continuity of a continuous circumferential band of hard dental tissue comprised of the buccal and palatal cusps attached together by intact marginal ridges is also a fundamental requirement to the structural integrity of posterior teeth. The ability of such structural continuum to resist the forces of occlusal loading is severely impaired by disruption of the continuum by the preparation of large cavity formations [5]. The cuspal fracture of maxillary premolars is a strong predisposing factor because of the anatomical structure, which has comparatively thin cusps and a small buccolingual dimension under functional loading [6, 7]. This structural weakness is even further increased with the further cut of the pulp chamber roof in the process of preparing endodontic access. Biomechanical effects of endodontic treatment have been under intense research and discussion. Previous views have blamed desiccation and greater brittle of the remaining dentin as the main causes of the increased fragility of pulpless teeth to fracture. Modern evidence indicates that the moisture level of endodontically treated dentin is merely slightly less than that of the vital dentin as well as that the mechanical traits of the dentin substrate itself do not essentially differ as a result of devitalization [8, 9]. Instead, fracture resistance has been found to largely depend on the amount and distribution of sound tooth structure which is the most significant loss of structural competence [10, 11].

The choice of restorative materials on the ultimate rehabilitation of affected endodontically treated teeth has undergone significant changes during the last few decades. Although full-coverage cast restorations have long been considered as the gold standard in protecting weakened endodontically treated teeth, the method requires further tooth preparation, more time and high financial cost [12, 13]. This has led to increased interest in assessing direct intracoronal restorative materials as potential substitutes especially in the case of the premolar teeth where economic and periodontal factors support a less invasive form of treatment. The modern restorative materials that could be used to this end would be the dual-cure composite core build-up materials, glass ionomer cements, dental amalgam and a combination of both through sandwich configurations. Multicore Flow is a dual-cure composite material, which has the theoretical benefits of chemical bonding to tooth structure, desirable mechanical properties such as large filler content and flexibility between self-curing and light-curing polymerization processes [14]. Glass ionomer cements such as Ketac Molar also offer the added benefit of being chemically bonded to enamel and dentin with a coefficient of thermal expansion that is comparable to the tooth structure itself, release of fluoride, yet their mechanical properties have long been rated as low when compared to composite resins [15, 16]. Although dental amalgam has a long history of clinical use, which is more than 100 years long, it cannot form an adhesive bond to tooth structure and acts only by mechanical retention [17]. Combining the adhesive and biological benefits of glass ionomer cement and the better mechanical properties and esthetic of composite resin, the sandwich technique is an attempt to integrate the strengths of the two classes of materials into a single restoration [18]. Therefore, it is of interest to compare of fracture resistance of endodontically restored maxillary premolars with extensive mesio-occluso-distal cavity filled with Multicore Flow, Ketac Molar glass ionomer cement, amalgam and open sandwich technique and the description of fracture patterns and failure modes.

## Materials and Methods:

## Study design and specimen collection:

This *in vitro* experimental study was conducted in the Department of Conservative Dentistry and Endodontics in coordination with the materials testing facility. Sixty freshly extracted human maxillary first premolars were collected from the Department of Oral and Maxillofacial Surgery.

## Inclusion and exclusion criteria:

Teeth extracted for orthodontic purposes that were free of caries, cracks, fractures, restorations and developmental anomalies were included. Primary teeth, teeth with visible structural defects and teeth with evidence of prior endodontic treatment were excluded.

## Specimen preparation and storage:

Following extraction, all teeth were debrided of soft tissue remnants and calculus deposits using an ultrasonic scaler, immersed in 0.1% thymol solution for 48 hours for disinfection and subsequently stored in distilled water at room temperature. All experimental procedures were completed within three months of extraction. Teeth of comparable dimensions were selected by measuring buccolingual and mesiodistal crown widths, ensuring that mean dimensional differences between groups did not exceed 5%. Periapical radiographs were obtained to confirm the presence of two root canals in all specimens.

## Specimen mounting:

To simulate the periodontal ligament and alveolar bone support, root surfaces were marked 2 mm below the cemento-enamel junction. A light-body addition silicone impression material was applied to the root surface to simulate the periodontal ligament. Each tooth was then embedded in a self-curing acrylic resin block within plastic cylinders, with the acrylic extending to within 2 mm of the cemento-enamel junction.

## Group allocation:

Specimens were randomly allocated into six groups of ten teeth each:

[1] Group 1 (Intact Control): Sound teeth without any preparation or treatment.

[2] Group 2 (Unrestored): Extensive MOD cavity preparation with endodontic treatment; cavity left unrestored.

[3] Group 3 (Multicore Flow): MOD cavity and endodontic treatment followed by restoration with Multicore Flow dual-cure composite.

[4] Group 4 (Ketac Molar): MOD cavity and endodontic treatment followed by restoration with Ketac Molar glass ionomer cement.

[5] Group 5 (Amalgam): MOD cavity and endodontic treatment followed by restoration with dental amalgam.

[6] Group 6 (Sandwich Technique): MOD cavity and endodontic treatment followed by restoration using the open sandwich technique with Ketac Molar and nanohybrid composite resin.

## Cavity preparation:

All teeth except Group 1 received standardized MOD cavity preparations with flat pulpal floors and no proximal steps. The buccolingual width of the occlusal isthmus and proximal boxes was established at one-half of the intercuspal width to simulate extensive structural compromise. The cavity floor was positioned 1 mm coronal to the cemento-enamel junction, achieving a total cavity depth of 5-6 mm measured from the cavosurface margin of the palatal cusp. All cavosurface margins were prepared at 90 degrees and consistency was ensured through parallel preparation of the facial and palatal cavity walls.

## Endodontic treatment:

Endodontic access cavities were prepared using dedicated access burs. Root canals were negotiated with stainless-steel K-files (sizes 10 and 15) and instrumented using rotary nickel-titanium files with a crown-down technique to a standardized apical preparation size of F1. Canals were obturated with matching gutta-percha points and a resin-based sealer to avoid the inhibitory effects of eugenol- containing sealers on composite polymerization.

## Restorative procedures:

Group 3: A dentin bonding agent was applied and light-cured. Multicore Flow was dispensed directly into the cavity using the intra-oral tip and polymerized from the occlusal surface for 20 seconds.

Group 4: Ketac Molar was proportioned, hand-mixed according to the manufacturer's instructions and placed into the prepared cavity using appropriate instruments.

Group 5: Amalgam alloy and mercury were triturated and the resulting amalgam was condensed incrementally into the prepared cavity.

Group 6: Ketac Molar was placed as a base layer of approximately 1 mm thickness. A universal adhesive was applied to the glass ionomer and cavity wall surfaces and light-cured for 10 seconds. Nanohybrid composite resin was then placed incrementally and light-cured. All restored specimens were stored in distilled water at 37°C for 24 hours prior to mechanical testing.

## Mechanical testing:

Specimens were secured in the testing jig of a computer-controlled universal testing machine. A steel bar was positioned at the center of the occlusal surface, oriented parallel to the long axis of the tooth and contacting the slopes of both cusps. Compressive axial loading was applied at a crosshead speed of 0.5 mm/min until specimen fracture occurred. Maximum breaking loads were recorded in kilonewtons by the computer interface.

## Assessment of fracture type and mode:

Following fracture, all specimens were immersed in ink for 5 minutes to facilitate visualization of fracture lines. Fractures were categorized as favorable (fracture line above the cemento-enamel junction) or unfavorable (fracture line extending below the cemento-enamel junction into the radicular portion). The mode of failure in restored groups was assessed under stereomicroscopy at 20× magnification and classified as adhesive (failure at the tooth-restoration interface), cohesive (failure within the restorative material), or mixed.

## Statistical analysis:

Data were analyzed using statistical software (SPSS version 22.0). Descriptive statistics including means and standard deviations were calculated for each group. One-way analysis of variance was performed to assess overall differences among groups, followed by the least significant difference post-hoc test for pairwise comparisons. The level of statistical significance was set at p < 0.05.

## Results:

The mean fracture resistance values, standard deviations and percentage changes relative to intact and unrestored controls are presented in Table 1. Intact control teeth (Group 1) demonstrated the highest mean fracture resistance (94.37 ± 1.39 KN), while unrestored endodontically treated teeth (Group 2) exhibited the lowest values (31.68 ± 6.20 KN), representing a 66.43% reduction in strength compared to intact teeth. Among the restored groups, Multicore Flow (Group 3) demonstrated the highest mean fracture resistance (72.07 ± 5.25 KN), followed by Ketac Molar (Group 4) at 60.59 ± 6.32 KN, sandwich technique (Group 6) at 37.30 ± 5.15 KN and amalgam (Group 5) at 34.04 ± 6.65 KN. The percentage increase in fracture resistance relative to unrestored teeth was greatest for Multicore Flow (56.04%); followed by Ketac Molar (47.71%), sandwich technique (15.06%) and amalgam (6.94%). One-way ANOVA revealed a statistically highly significant difference in mean fracture resistance among all groups (F = 106.55, p < 0.001). Post-hoc analysis using the least significant difference test demonstrated specific pairwise relationships as presented in Table 2. The distribution of fracture types and failure modes is presented in Table 3. Intact control teeth predominantly exhibited favorable fractures (80%), while all unrestored teeth demonstrated unfavorable fractures (100%). Among restored groups, the majority of specimens in all groups showed unfavorable fracture patterns. Multicore Flow and Ketac Molar groups did not demonstrate identifiable adhesive or cohesive failure modes, suggesting integrated failure involving both tooth and restorative material. Amalgam restorations showed predominantly mixed failure (80%), while sandwich technique restorations exhibited predominantly adhesive failure (70%). No statistically significant differences were observed between intact teeth and teeth restored with Multicore Flow or Ketac Molar. Similarly, no significant differences existed between unrestored teeth and those restored with amalgam or sandwich technique.

## Discussion:

This study results indicate that the strength of the endodontically restored premolar with large mesio-occluso-distal cavity preparations is profoundly and statistically significantly affected by the selection of the restorative material and thus it is true that the post-endodontic restoration is a material-dependent clinical decision with a direct impact on the long term survival of the tooth. The fact that the fracture resistance has dramatically decreased after a long time cavity preparation and endodontic procedure where 66.43 percent decrease was taken compared to intact teeth supports the previous studies which have shown that there is a severe structural weakening that occurs in connection with the loss of marginal ridges, pulp chamber roof and the loss in the number of supporting dentin [19, 20]. Such a scale of structural concession should highlight the extreme significance of the restorative step as an inherent part of endodontic treatment and not a distinct and secondary factor. The earlier biomechanical evaluations revealed that marginal ridges integrity is a significantly bigger contributor to the overall tooth toughness compared to pulp chamber roof and that the joint loss of the two structures yields an additive impact on weakening effect that is enormous in relation to increasing the likelihood of fractures [21, 22]. The high functionality of Multicore Flow that resulted in a restoration of the tooth strength of about 76 percent of the original and no statistically significant difference was observed with intact teeth is explained by a variety of material-specific factors. The dual-cure polymerization process guarantees full conversion in the entire bulk of the restoration even in the deep cavity preparations where the curing lights could not reach [23]. The presence of high filler content with a particle size of between 0.04 and 25 micrometers ensures high compressive and flexural strength whereas the flowable consistency enables the tooth-restoration complex to be well adapted to the cavity walls, reducing the formation of voids and improving the integrity of the tooth-restoration complex [24]. The results can be related to previous studies that show that dual-cure composite core materials can be integrated to residual tooth structure that is effective in redistributing the occlusal stresses over the restored tooth [25, 26]. Ketac Molar glass ionomer cement also showed a good performance with the restoration of about 64 percent of the original tooth strength that is not significantly different to the intact teeth. The large powder to liquid ratio of this formulation forms a highly compressed particle network of glasses that gives the formulation very high compressive strength and wears resistance [27]. Diagnostic chemical bonding of glass ionomer cements to enamel and dentin using ionic bonding with calcium and phosphate ions in the hydroxyapatite structure forms a real physicochemical unity in the tooth-restoration interface and could play a role in the transfer of stress and structural reinforcement [28,29]. The fact that no adhesive or cohesive failure can be identified in this group is another indicator of the idea of integrated structural behavior between the restoration and the remaining tooth structure. The significantly lower performance of amalgam, which gave only 6.94% improvement over unrestored teeth and no statistically significant difference with the unrestored group is a clinically consequential finding. Dental amalgam is hydrophobic in nature with no chemical bond formation with hydrophilic tooth structure which can only be held by mechanical means in the prepared cavity [30, 31]. During compressive loading, in the absence of a bonded interface, the restoration is able to move independently of the tooth structure, where the amalgam is not a unifying structural component, but instead a rigid wedge between the buccal and palatal cusps. Previous studies have proved this merging system to be the main cause of cuspal fracture in teeth that were restored with non-adhesive intracoronal restorations [32, 33]. The mixed mode of failure, which is mostly evident in the amalgam group, the combination of adhesive separation at the tooth-restoration interface and cohesive fracture, is in line with the complex patterns of stresses produced by non-bonded rigid restoration in a weakened tooth. Although theoretically, the sandwich method shows the benefit of integrating the adhesive strength of glass ionomer cement with the mechanical strength of composite resin, practical experiment revealed a rather low increase on fracture resistance. The largely adhesive type of failure that happened in this group which was at the interface between the glass ionomer base and the overlaying composite indicates poor bonding between two layers of the materials [34]. Also, the contraction stresses caused by polymerization contraction of the composite resin component can result in adaptation of the adhesive interface to the cavity walls and structural enhancement capacity of the restoration [35, 36]. Placement of a horizontal fiber post before restoration may increase the incidence of unfavorable fractures in restored teeth [37, 38]. These observations indicate that the open sandwich method as implemented in this paper does not give the material property synergies that its theoretical basis promises. The fracture type analysis indicated that the vast majority of specimens in all groups with a single exception of intact controls had an unfavorable fracture pattern below the cemento-enamel junction. This observation has serious clinical implications because subcrestal fissures usually make teeth unrestorable without further surgical treatment or extractions are required. The fact that unfavorable fractures are very common even in groups that show relatively high fracture resistance values indicates that some restorative materials can significantly raise the amount of force that is needed to cause fracture but they may not necessarily change the fracture propagation pathway, once the process of fracture has started. There are a number of weaknesses of this study that should be mentioned. Although the in vitro design allows standardization of the experimental conditions, it fails to mimic the complex intraoral conditions of cyclic fatigue loading, thermal changes and changes in moisture as well as the progressive erosion of adhesive interfaces with time. The use of one static compressive loading to catastrophic failure does not mimic the repetitive subcritical loading which often precedes clinical fracture. This could overestimate the long-term clinical outcome of the adhesive restorative materials because of the lack of thermocycling and fatigue protocols. Also, the sample of ten specimens per group might have been comparatively small to statistically reveal the existence of minor difference between some of the groups.

## Conclusion:

Endodontic treatment with extensive MOD cavity preparation significantly reduces fracture resistance of maxillary premolars, necessitating appropriate restorative reinforcement. Dual-cure composite and glass ionomer cement demonstrated superior fracture resistance comparable to intact teeth, whereas amalgam and sandwich technique offered minimal reinforcement. Adhesive restorative materials are more effective in strengthening weakened teeth and may serve as suitable interim restorations before definitive cuspal coverage.

## Figures and Tables

**Table 1:** Mean fracture resistance values and percentage changes for all experimental groups

**Group**	**Material**	**n**	**Mean ± SD (KN)**	**Reduction from Intact (%)**	**Increase from Unrestored (%)**
1	Intact control	10	94.37 ± 1.39	-	-
2	Unrestored (prepared + RCT)	10	31.68 ± 6.20	66.43	-
3	Multicore Flow	10	72.07 ± 5.25	23.63	56.04
4	Ketac Molar GIC	10	60.59 ± 6.32	35.8	47.71
5	Amalgam	10	34.04 ± 6.65	63.93	6.94
6	Sandwich technique	10	37.30 ± 5.15	60.48	15.06
RCT = root canal
treatment; GIC = glass
ionomer cement

**Table 2:** Post-Hoc pairwise comparisons (LSD Test) between all experimental groups

**Comparison**	**Mean Difference(KN)**	**p Value**	**Significance**
Group 1 vs Group 2	62.69	<0.001	Significant
Group 1 vs Group 3	22.3	0.804	Not significant
Group 1 vs Group 4	33.78	0.071	Not significant
Group 1 vs Group 5	60.33	<0.001	Significant
Group 1 vs Group 6	57.08	<0.001	Significant
Group 2 vs Group 3	-40.39	<0.001	Significant
Group 2 vs Group 4	-28.91	<0.001	Significant
Group 2 vs Group 5	-2.36	0.355	Not significant
Group 2 vs Group 6	-5.62	0.5	Not significant
Group 3 vs Group 4	11.48	0.345	Not significant
Group 3 vs Group 5	38.03	<0.001	Significant
Group 3 vs Group 6	34.77	<0.001	Significant
Group 4 vs Group 5	26.55	<0.001	Significant
Group 4 vs Group 6	23.29	<0.001	Significant
Group 5 vs Group 6	-3.26	0.314	Not significant

**Table 3:** Distribution of fracture types and failure modes across experimental groups

**Group**	**Material**	**Favorable n (%)**	**Unfavorable n (%)**	**Adhesive n (%)**	**Cohesive n (%)**	**Mixed n (%)**
1	Intact control	8 (80)	2 (20)	-	-	-
2	Unrestored	0 (0)	10 (100)	-	-	-
3	Multicore Flow	2 (20)	8 (80)	0	0	0
4	Ketac Molar	1 (10)	9 (90)	0	0	0
5	Amalgam	1 (10)	9 (90)	2 (20)	0	8 (80)
6	Sandwich technique	1 (10)	9 (90)	7 (70)	3 (30)	0
